# Supplementary material for: Structure of the Deactive State of Mammalian Respiratory Complex I
Source: Structure. 2018 Feb 6;26(2):312–319.e3. doi: 10.1016/j.str.2017.12.014 (PMC5807054; doi:10.1016/j.str.2017.12.014)
Supplement: Document S1. Figures S1–S5 and Tables S1–S3 [file mmc1.pdf]

Structure, Volume *26*

Supplemental Information

Structure of the Deactive

State of Mammalian Respiratory Complex I

James N. Blaza, Kutti R. Vinothkumar, and Judy Hirst

Supplemental Information:

Structure of the deactive state of mammalian respiratory complex I

James N. Blaza[1], Kutti R. Vinothkumar[2] and Judy Hirst[1]

Supplemental Figure 1. Related to Figure 2.

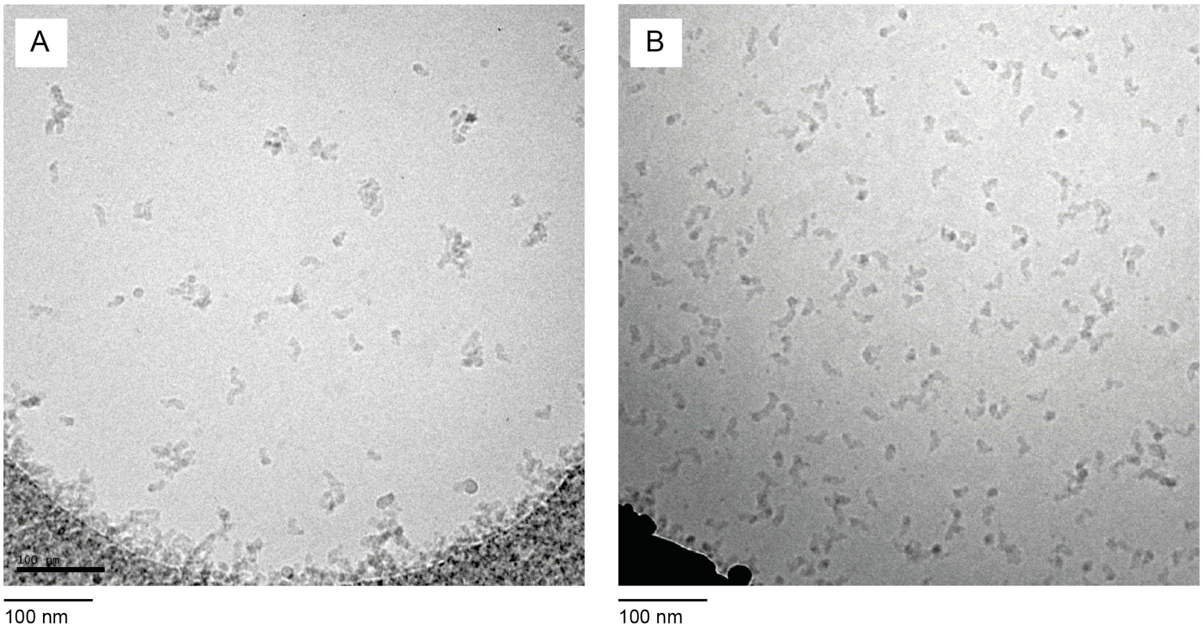

Micrographs from a sample of deactive complex I imaged on Quantifoil and PEGylated gold UltrAuFoil grids. A) Micrograph from a representative hole in a Quantifoil (0.6/1) grid that had been glow-discharged for 90 s at 20 mA. B) Micrograph from a representative hole in a PEGylated gold (1.2/1.3) grid prepared as described in Methods. Both grids were prepared using the same deactive complex I preparation, which had been frozen before grid preparation, using a Vitrobot (blot force -10, temperature 4 °C, and relative humidity 100%) but with longer blotting time (8 s) for the PEGylated gold grids than the Quantifoil grids (2 s) to account for their increased hydrophilicity. Both grids were imaged in an FEI T12 microscope with substantial defocus.

Supplemental Figure 2. Related to Figure 2.

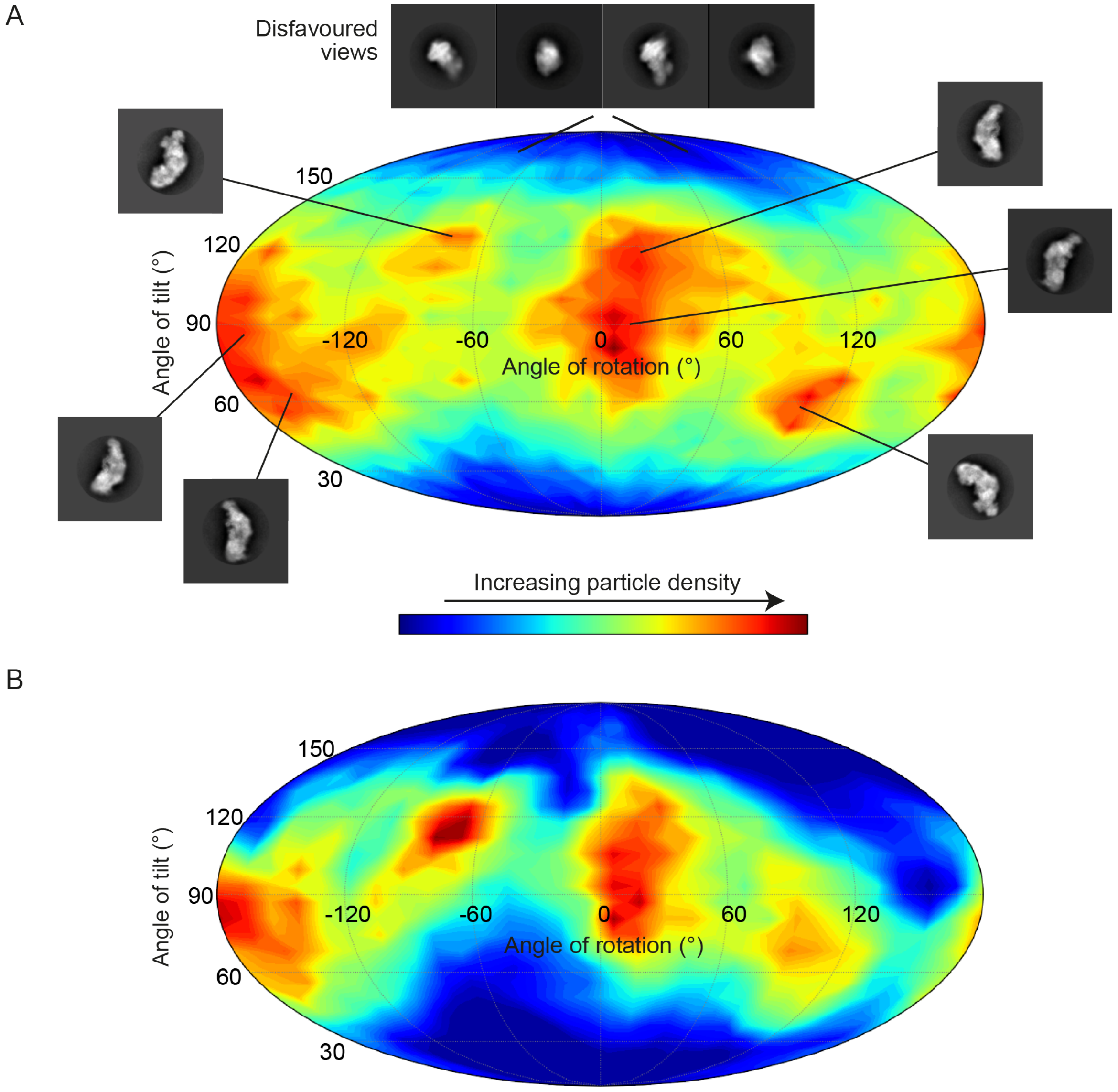

Analysis of the orientation of deactive complex I particles on PEGylated gold UltrAuFoil and Quantifoil grids. A) Distribution of particle orientations on PEGylated 0.6/1 gold grids with example 2D class averages from different regions of the plot. B) Particle orientations on 0.6/1 Quantifoil grids from a previously reported dataset (Zhu et al., 2016). The PEGylated gold dataset contains 125,007 particles, and the Quantifoil dataset 115,974 particles; each dimension

was split into 30 bins for calculation of particle densities. The angles of rotation and tilt are taken from the '_rlnAngleRot' and '_rlnAngleTilt' values for each particle after autorefinement in RELION 1.4. As expected there was no correlation with the in-plane rotation (_rlnAnglePsi) parameter.

Supplemental Figure 3. Related to Figure 3.

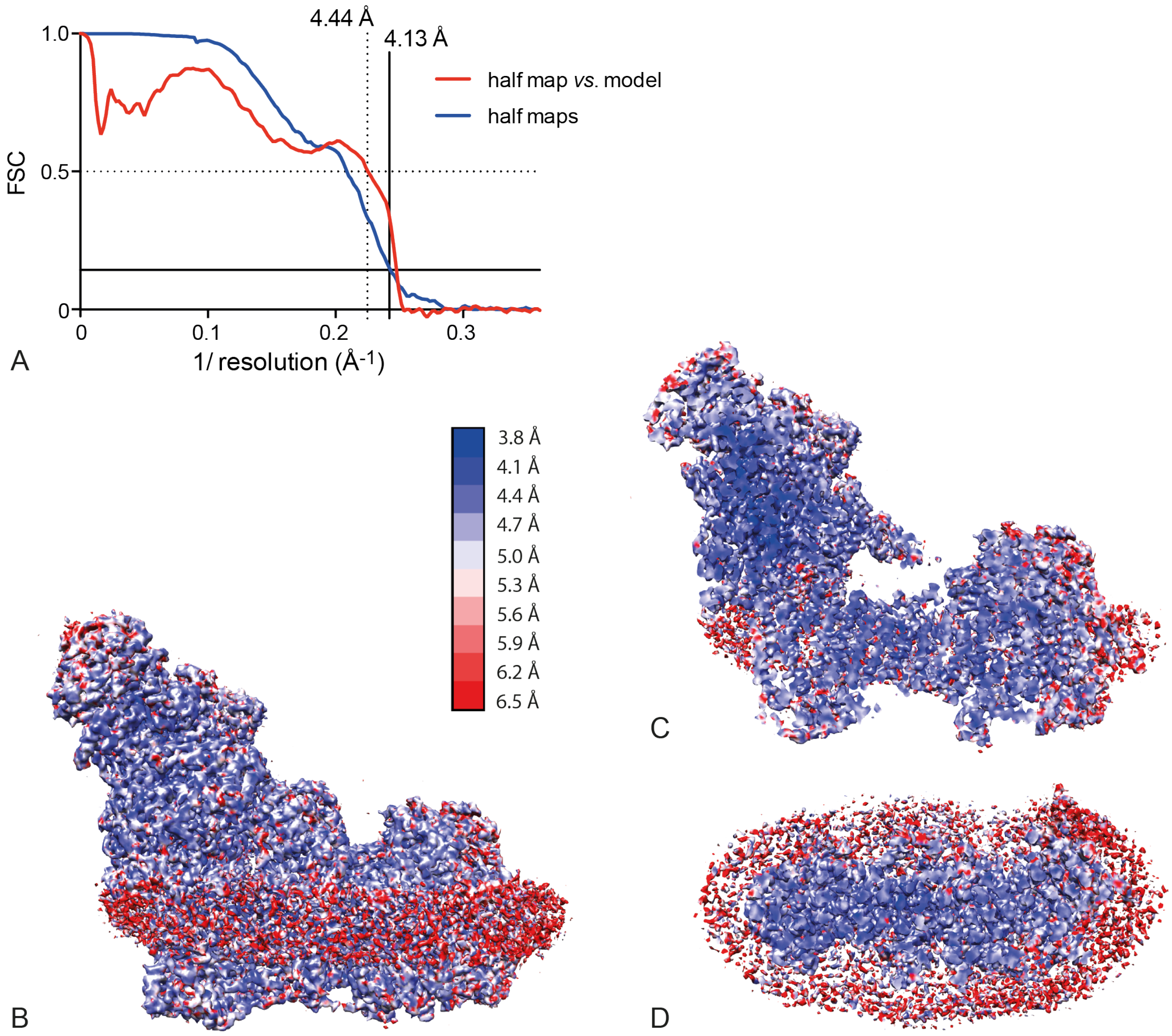

Resolution estimation and ResMap analysis for deactive complex I. A) FSC curve for the dominant class of the deactive preparation. B-D) Local resolution for the dominant class of the deactive preparation analyzed using ResMap (Kucukelbir et al., 2014). In panel C the resolution of the inner core of the complex, particularly around the iron-sulfur clusters, is shown to have a higher resolution than the outside (panel B); the amorphous detergent/phospholipid belt is poorly resolved. Panel D shows a cross-section of the complex from the matrix side, with the hydrophilic domain cut away.

Supplemental Figure 4. Related to Figure 3.

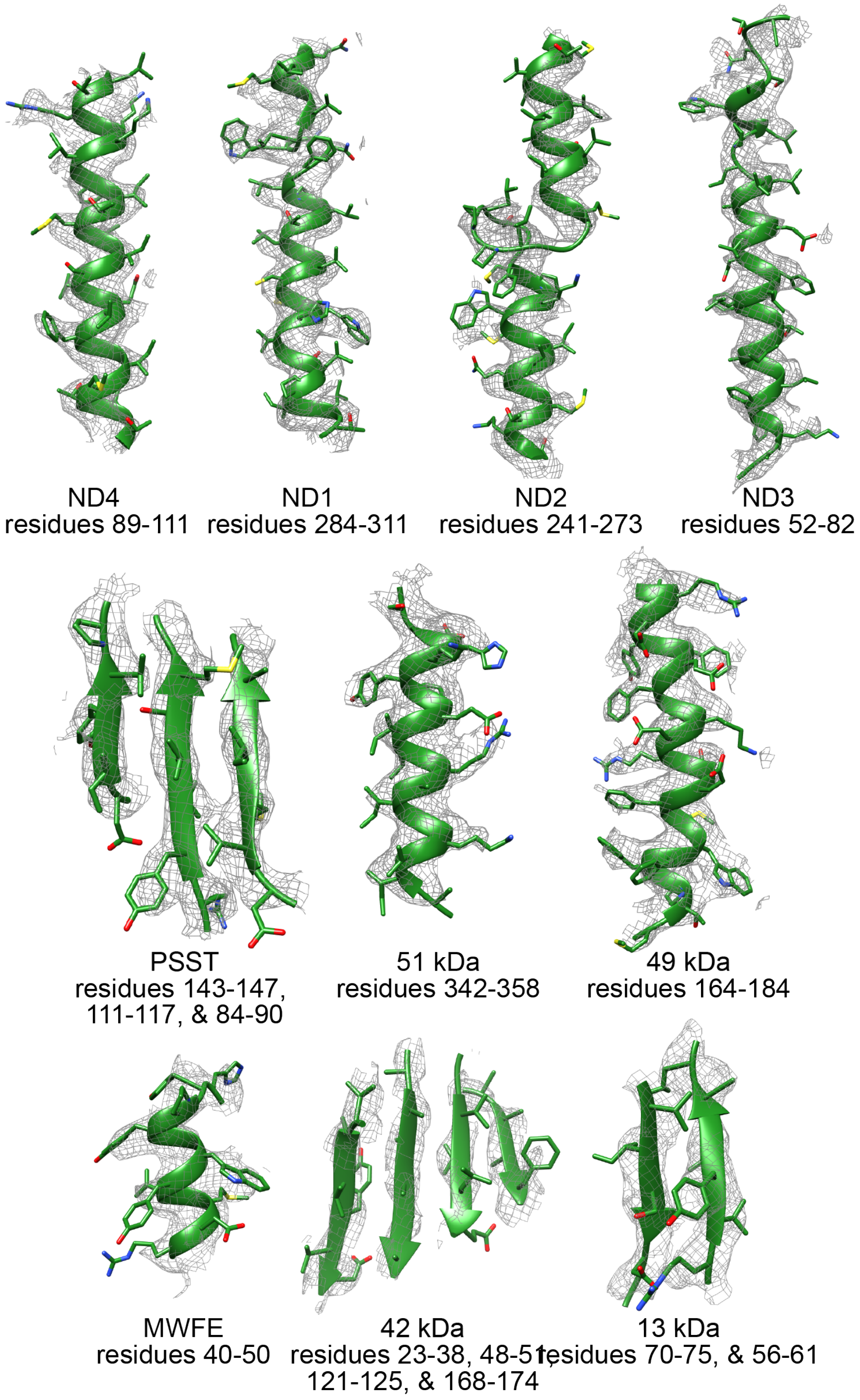

Example regions of the cryoEM density map for deactive complex I, and the model fitted to the map.

Supplemental Figure 5. Related to Table 1.

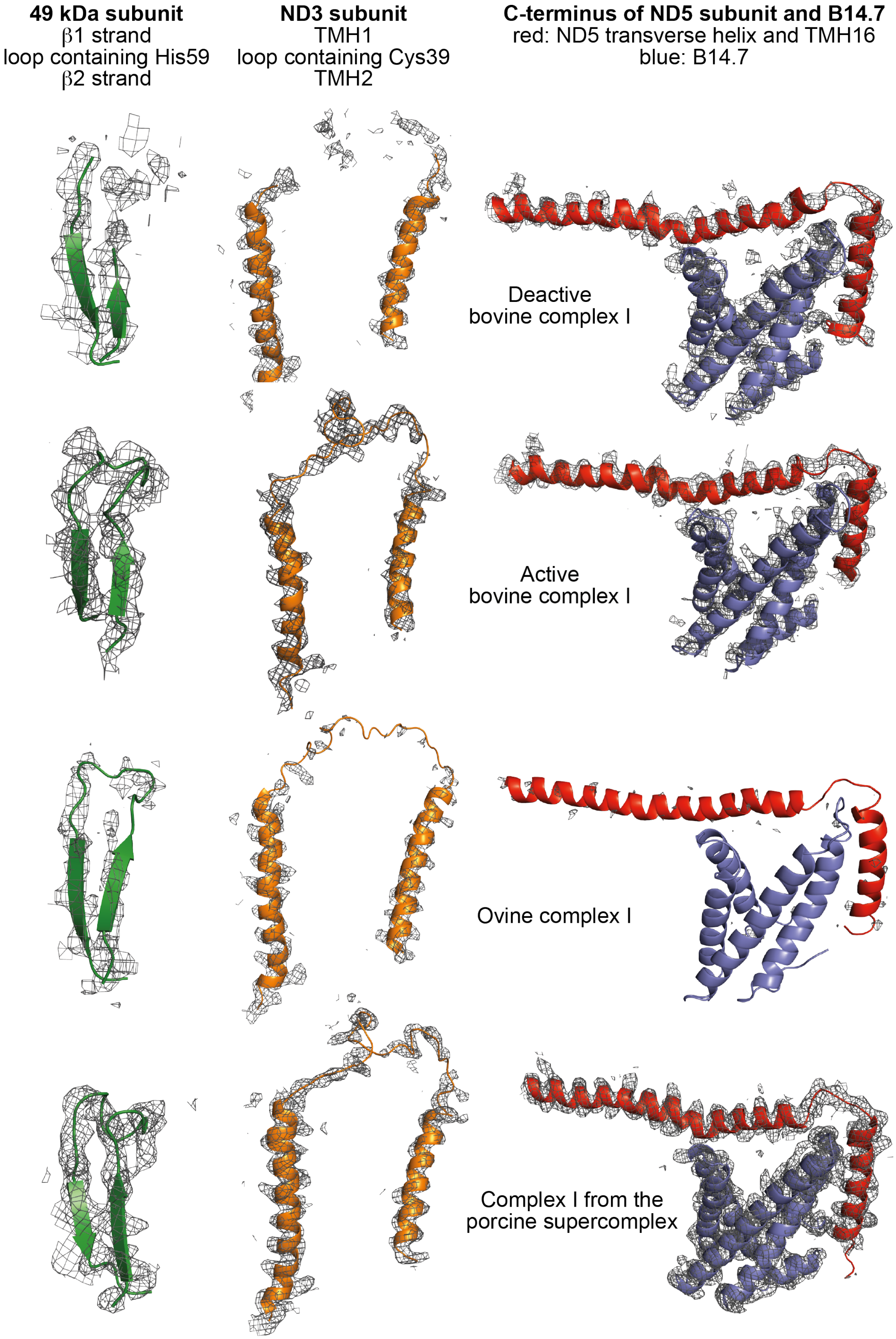

Comparison of characteristic densities in deactive and active bovine complex and in structures from other mammalian species. Green: comparison of densities for the β1-β2 loop in the 49 kDa (NDUFS2) subunit. Orange: comparison of densities for the TMH1-2 loop in subunit ND3. Red: comparison of densities for the C-terminus of subunit ND5 and blue: subunit B14.7 (NDUFA11). The contour levels for the density were set in Pymol to appropriate levels for the TMHs of subunit ND3, and then kept constant for the other subunits (deactive bovine complex I, RMSD value of 6.3; active bovine complex I, 6.8; ovine complex I, 6.0; porcine supercomplex, 8.5). A carve radius of 2.5 Å was used throughout. The deactive bovine complex structure is from the data described here; the densities for elements not modeled were carved using the structures of the subunits from the active complex. The active bovine complex structure is from 5LC5.pdb and EMD-4032 (Zhu et al., 2016). The ovine complex I structure is for the isolated complex I of Sazanov and coworkers (Fiedorczuk et al., 2016) (5LNK.pdb and EMD-4084). The porcine complex I structure is from the respirasome structure of Yang and coworkers (Wu et al., 2016) (5GUP.pdb and EMD-9539).

Supplemental Table 1- Data collection, refinement, and model statistics for the dominant class of the deactive enzyme. Related to STAR Methods.

**Data Collection**	
Pixel size (Å)	1.38
Defocus range (μm)	1.3-3.1
Voltage (kV)	300
Number of particles in refinement	109,591
**Map refinement**	
Resolution (Å)	4.13
B-factor used for map sharpening ($Å^2$)	-110
**Model statistics**	
Non-hydrogen atoms	52,698
Protein residues	7,811
% of total	91.7
Core subunit residues	4,297
% of total	95.6
Supernumerary subunit residues	3,514
% of total	87.4
Average B-factor ($Å^2$)	78.7
**RMS deviations**	
Bonds (Å)	0.009
Angles (°)	1.534
**Validation**	
Molprobity score	1.92
Clashscore, all atoms	2.56
**Ramachandran plot**	
Favored (%)	87.5
Outliers (%)	2.6

Supplemental Table 2- Summary of the models for the core subunits of deactive bovine complex I. Related to STAR Methods.

Subunit	Other names*	Chain	Total residues	Modelled residues	Assigned residues	Unknown residues	% residues modelled	% residues assigned	% with sidechains	% unknown residues
ND1	Nqo8<br>NuoH	H	318	3-200<br>218-315	3-200<br>218-315	-	93.1	93.1	90.3	0
ND2	Nqo14<br>NuoN	N	347	2-345	2-345	-	99.1	99.1	87.3	0
ND3	Nqo7<br>NuoA	A	115	2-27<br>51-112	2-27<br>51-112	-	76.5	76.5	72.2	0
ND4	Nqo13<br>NuoM	M	459	3-459	3-459	-	99.6	99.6	93.0	0
ND4L	Nqo11<br>NuoK	K	98	2-96	2-96	-	96.9	96.9	96.9	0
ND5	Nqo12<br>NuoL	L	606	2-605	2-605	-	99.7	99.7	86.1	0
ND6	Nqo10<br>NuoJ	J	175	2-172	2-172	-	97.7	97.7	80.0	0
75 kDa	NDUFS1<br>Nqo3<br>NuoG	G	704	6-693	6-693	-	97.7	97.7	29.0	0
51 kDa	NDUFV1<br>Nqo1<br>NuoF	F	444	14-438	14-438	-	95.7	95.7	18.2	0
49 kDa	NDUFS2<br>Nqo4<br>NuoCD	D	430	5-50<br>61-430	5-50<br>61-430	-	96.7	96.7	87.0	0
30 kDa	NDUFS3<br>Nqo5<br>NuoCD	C	228	10-213	10-213	-	89.5	89.5	86.0	0
24 kDa	NDUFV2<br>Nqo2<br>NuoE	E	217	8-193	8-193	-	85.7	85.7	1.8	0
PSST	NDUFS7<br>Nqo6<br>NuoB	B	179	27-173	27-173	-	82.1	82.1	82.1	0
TYKY	NDUFS8<br>Nqo9<br>NuoI	I	176	1-176	1-176	-	100	100	92.0	0

*The names of the human, *T. thermophilus* and *E. coli* subunits (if different to the names in *B. taurus*).

Supplemental Table 3- Summary of the models for the supernumerary subunits of deactive bovine complex I. Related to STAR Methods.

Subunit	Human name	Chain	Total residues	Modelled residues	Assigned residues	Unknown residues	% residues modelled	% residues assigned	% with sidechains	% unknown residues
42 kDa	NDUFA10	O	320	5-318	5-318	-	98.1	98.1	26.6	0
39 kDa	NDUFA9	P	345	2-186<br>200-252<br>280-324	-	2-186<br>200-252<br>280-324	82.0	0	0	82.0
18 kDa	NDUFS4	Q	133	11-133	11-133	-	91.7	92.5	0	0
15 kDa	NDUFS5	e	105	6-94	6-94	-	84.8	84.8	42.9	0
13 kDa	NDUFS6	R	96	1-93	1-93	-	96.9	96.9	34.3	0
10 kDa	NDUFV3	s	75	34-74	34-74	-	54.7	54.7	0	0
AGGG	NDUFB2	j	72	8-59	-	8-59	72.2	0	0	72.2
ASHI	NDUFB8	l	158	5-122	-	5-122	74.7	0	0	74.7
ESSS	NDUFB11	g	125	25-121	25-121	-	77.6	77.6	41.6	0
KFYI	NDUFC1	c	49	1-46	1-46	-	93.9	93.9	59.2	0
MNLL	NDUFB1	f	57	3-56	3-56	-	94.7	94.7	42.1	0
MWFE	NDUFA1	a	70	1-64	1-64	-	91.4	91.4	71.4	0
PDSW	NDUFB10	p	175	4-172	76-142	4-75<br>143-172	96.6	38.3	30.9	58.3
PGIV	NDUFA8	X	171	5-168	5-168	-	95.9	95.9	53.8	0
SDAP-α	NDUFAB1	T	88	8-82	8-82	-	85.2	85.2	0	0
SDAP-β	NDUFAB1	U	88	4-88	4-88	-	96.6	96.6	0	0
SGDH	NDUFB5	h	143	7-140	7-140	-	93.7	93.7	21.7	0
B22	NDUFB9	n	178	10-175	10-175	-	93.3	93.3	36.0	0
B18	NDUFB7	o	136	57-114	57-114	-	42.6	42.6	2.9	0
B17.2	NDUFA12	q	145	2-139	2-139	-	95.2	95.2	0	0
B17	NDUFB6	i	127	6-32<br>40-118	6-32	40-118	83.5	21.3	14.2	62.2
B16.6	NDUFA13	Z	143	5-141	5-98	99-141	95.8	65.7	47.6	30.1
B15	NDUFB4	m	128	11-128	11-128	-	92.2	92.2	67.2	0
B14.7	NDUFA11	Y	140	1-138	1-138	-	98.6	98.6	98.6	0
B14.5a	NDUFA7	r	112	1-69<br>93-111	-	-	78.6	78.6	0	0
B14.5b	NDUFC2	d	120	4-116	4-97	98-116	94.2	78.3	52.5	15.8
B14	NDUFA6	W	127	16-126	16-126	-	87.4	87.4	57.5	0
B13	NDUFA5	V	115	8-113	8-113	-	92.2	92.2	40.9	0
B12	NDUFB3	k	97	16-89	-	16-89	76.3	0	0	76.3
B9	NDUFA3	b	83	1-80	1-45	46-80	96.4	54.2	54.2	42.2
B8	NDUFA2	S	98	16-95	16-95	-	81.6	81.6	0	0
